# Salicylic and jasmonic acid crosstalk manipulation by whiteflies weakens larval parasitoid recruitment

**DOI:** 10.1093/jxb/eraf462

**Published:** 2026-01-12

**Authors:** Pablo Urbaneja-Bernat, Víctor Flors

**Affiliations:** Plant Immunity and Biochemistry Group, Biology, Biochemistry and Natural Sciences Department, Universitat Jaume I, Castellón, Spain; Plant Immunity and Biochemistry Group, Biology, Biochemistry and Natural Sciences Department, Universitat Jaume I, Castellón, Spain

**Keywords:** Biocontrol, herbivore-induced plant volatiles, hormonal crosstalk, jasmonic acid, multitrophic interactions, NPR1, parasitoids, salicylic acid, whiteflies

## Abstract

This article comments on:

**Dong Y-M, Sang Y-L, Wang S-Z, Turlings TCJ, Li Y-H, Xue D-W, Zhang P-J.** 2025. Interference with phytohormone signaling by whiteflies differentially affects plant attractiveness to a larval and an egg parasitoid of the cabbage white butterfly. Journal of Experimental Botany **76**, 4615–4626. https://doi.org/10.1093/jxb/eraf208

This article comments on:

**Dong Y-M, Sang Y-L, Wang S-Z, Turlings TCJ, Li Y-H, Xue D-W, Zhang P-J.** 2025. Interference with phytohormone signaling by whiteflies differentially affects plant attractiveness to a larval and an egg parasitoid of the cabbage white butterfly. Journal of Experimental Botany **76**, 4615–4626. https://doi.org/10.1093/jxb/eraf208

**Interactions between signaling hormones act as a central hub of plant defenses, determining how plants respond to attacks by different herbivores and pathogens. Understanding how this hormonal crosstalk influences direct and indirect defenses is key for both ecological insight and pest management. Dong *et al*. (2025) reveal that the phloem-feeding whitefly *Bemisia tabaci* activates salicylic acid (SA) signaling, which suppresses jasmonic acid- (JA) dependent volatiles, thereby weakening the attraction of larval parasitoids of *Pieris rapae* while leaving recruitment of egg parasitoids unaffected. By linking NPR1-mediated SA–JA antagonism with changes in herbivore-induced volatiles and parasitoid behavior, this study provides mechanistic clarity on how herbivores affect plant defenses and reshape multitrophic interactions.**

## Plants under multiple attacks

In agroecosystems, plants are rarely attacked by a single herbivore, and face simultaneous pressures from chewing and sucking insects, such as larvae, sap-feeding insects, mites, or pathogens. To manage these attacks, plants use a defense regulated by hormonal signaling. The JA pathway is typically associated with responses to chewing herbivores, while the SA pathway is central against sucking herbivores (Erb and Reymond, 2019). Plants have evolved plastic responses though hormonal crosstalk, saving energy to reduce defense–growth trade-offs. Reasonably, in the evolutionary arms race, some insects have evolved mechanisms to interfere with and manipulate host hormonal responses. Although SA/JA crosstalk can be both synergistic and antagonistic, there is evidence of negative functional outcomes during pest co-infestations (Al-Zahrani *et al*., 2020), since activation of SA can antagonize JA responses (Koornneef and Pieterse, 2008). These negative hormonal interactions may affect direct plant responses by repressing defenses against the attacker and also interfere with the plant interaction at the ecological and multitrophic level, since herbivore-induced plant volatiles (HIPVs) increase this complexity. These blends/odors not only deter herbivores but also act as indirect defenses by attracting natural enemies (Turlings and Erb, 2018). When multiple herbivores attack simultaneously, HIPV emission can be silenced, enhanced, or altered depending on the species combination (Ponzio et al., 2013; Cusumano *et al*., 2015). Moreover, insect oviposition is now recognized as a defense trigger in its own right, with egg deposition inducing specific signaling cascades and HIPVs that recruit egg parasitoids (Hilker and Fatouros, 2015). An important gap is how hormonal signaling supports these variable outcomes and how this affects recruitment of the third trophic level.

## Whiteflies as stealth manipulators

**Fig. 1.**
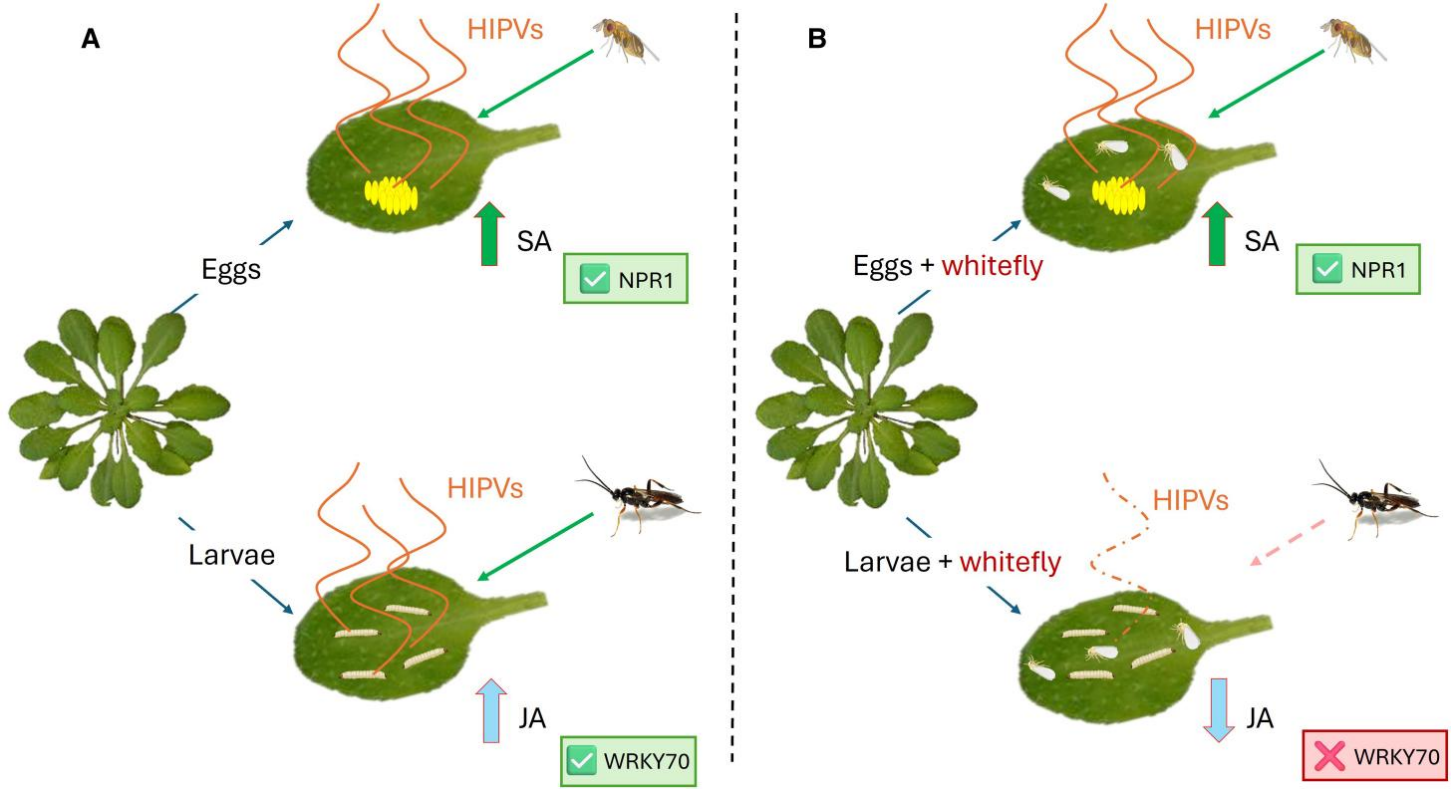
Conceptual model of whitefly interference in Arabidopsis indirect defenses. In Arabidopsis, oviposition and larval feeding by *Pieris* activate distinct hormonal pathways that regulate indirect defenses. (A) Egg deposition induces salicylic acid (SA) signaling, leading to the emission of herbivore-induced plant volatiles (HIPVs) that attract egg parasitoids (*Trichogramma chilonis*). Larval feeding activates jasmonic acid (JA) signaling, which promotes HIPVs that recruit larval parasitoids (*Cotesia rubecula*). (B) Feeding by the whitefly *Bemisia tabaci* interferes with this system by overstimulating SA signaling. This antagonizes JA through NPR1-dependent crosstalk, independently of WRKY70. Egg-induced HIPVs persist, ensuring egg parasitoid recruitment, while larval feeding weakens HIPV emission and reduces larval parasitoid attraction.

Beyond direct feeding and virus transmission, *Bemisia tabaci* (Hemiptera: Aleyrodidae) manipulates host defenses by inducing SA responses. Recently, several white fly protein effectors such as BtE3 and Bt56 were described to activate the SA pathway (Xu *et al*., 2018; Peng *et al*., 2023). One of the main hypotheses for SA boosting is the antagonistic effect over JA-dependent responses, thereby weakening resistance to chewing herbivores (Zarate *et al*., 2007). Dong *et al*. (2025) investigated the consequences of this interference for tritrophic interactions involving a co-infestation experiment, using *Arabidopsis thaliana* as a model and the specialist herbivore *Pieris rapae* L. (Lepidoptera: Pieridae) at egg and larval stages. At the third trophic level they used the egg parasitoid *Trichogramma chilonis* (Hymenoptera: Trichogrammatidae) and the larval parasitoid *Cotesia rubecula* (Hymenoptera: Braconidae). They demonstrated that co-infestation with *B. tabaci* reduced larval but not egg parasitoid attraction. Molecular analyses showed that JA suppression was mediated by NPR1 rather than WRKY70, and greenhouse assays validated their results in a crucifer system with *Brassica oleracea*, confirming these non-reciprocal effects under more natural conditions. Thus, *B. tabaci* exploits NPR1-mediated SA signaling to suppress JA-dependent HIPVs (Pieterse and van Loon, 2004), weakening the recruitment of larval parasitoids while preserving egg parasitoid efficacy. By combining molecular genetics, chemical ecology, and behavioral assays in both laboratory and greenhouse settings, this study provides a comprehensive view of how *B. tabaci* can manipulate plant defenses (Fig. 1).

## Ecological implications

The ecological message is that *B. tabaci* does not indiscriminately disrupt indirect defenses but affects the parasitic wasp asymmetrically, reducing larval parasitoid attraction, while leaving egg parasitoid attraction unchanged. This non-reciprocal interaction has refined ecological consequences by hampering larval parasitoid attraction; whiteflies indirectly promote the survival of chewing herbivores that would otherwise be controlled by parasitoids. From the plant side, responses within multitrophic and multiway interactions remain poorly understood, as most studies still focus on two-way interactions addressing plant responses to a single attacker (Gruden *et al*., 2020).

Why might this matter for *B. tabaci* itself? One possibility is that the continued presence of larvae alters plant physiology in ways that benefit phloem feeders. Herbivory can manipulate phloem composition, for example by increasing sugar availability, as shown for brown planthoppers that exploit rice sugar transporters to enhance their own feeding performance (Yu *et al*., 2024). It can also alter defense-related proteins; in the case of *B. tabaci*, such proteins are overexpressed in the phloem sap, accumulate in honeydew, and have been linked to enhancing the fitness of the third trophic level (Urbaneja-Bernat *et al*., 2024), suggesting a potential trade-off that merits further investigation. Alternatively, the observed asymmetry could be a collateral outcome of the SA-based manipulation strategy, which primarily guarantees whitefly feeding but facilitates competitors. Either way, these findings highlight the capacity of herbivores to engineer community-level interactions by manipulating plant signaling. Whiteflies act not only as phloem feeders but also as ecological interferers that reshape multitrophic networks.

## Implications for integrated pest management

In integrated pest management (IPM), parasitoids are central allies. If whitefly outbreaks selectively compromise the effectiveness of larval parasitoids while leaving egg parasitoids unaffected, this asymmetry may trigger the variable success of the parasitoid-based control in whitefly-infested systems. Recognizing and counteracting such hormonal interference will be essential for developing more resilient IPM strategies. Future IPM strategies could include priming plants to sustain JA signaling, even under strong SA induction, that helps to rebalance hormonal pathways that herbivores can generate (Flors *et al*., 2024). In parallel, natural variation in wild relative crops offers valuable complementary traits. For example, in *Solanum chmielewskii*, the vitamin riboflavin in the phloem sap has been identified as a mobile factor that delays whitefly nymphal development, providing a direct defense independent of trichome metabolites. Such findings highlight the promise of combining indirect defenses mediated by HIPVs with direct metabolic resistance to counteract whitefly manipulation. Alternatively, breeding for plants with deregulated hormonal crosstalk may also open up interesting future tools for enhanced resistance during co-infestations. Harnessing both hormonal ecology and metabolomic diversity may ultimately strengthen IPM strategies and reduce dependence on pesticides.

## Ecological and evolutionary perspectives

This study demonstrates that hormones and HIPVs are not fixed signals but are dynamic traits open to disturbance and even manipulation, with herbivores such as *B. tabaci* modifying plant signaling and reshaping plant–parasitoid communication. These findings underscore the dynamic co-evolution among plants, herbivores, and natural enemies, with hormonal signaling itself becoming a crossroads of defense. Ecologically, they highlight the need to consider co-infestation and hormonal interference, which consequently affect HIPVs and multitrophic interactions. Such asymmetries further illustrate that herbivores can act as keystone species, shaping the evolutionary trajectory of plant defenses (Poelman and Kessler, 2016). More broadly, this work positions hormonal crosstalk as a central axis in both the ecology and IPM of plant–insect interactions.
